# Using Information Technology to Assess Patient Risk Factors in Primary Care Clinics: Pragmatic Evaluation

**DOI:** 10.2196/24382

**Published:** 2021-02-02

**Authors:** Leanne Kosowan, Alan Katz, Gayle Halas, Lisa LaBine, Alexander Singer

**Affiliations:** 1 Rady Faculty of Health Sciences University of Manitoba Winnipeg, MB Canada; 2 Manitoba Centre for Health Policy Winnipeg, MB Canada

**Keywords:** risk factors, information technology, primary health care, primary prevention

## Abstract

**Background:**

Tobacco use, physical inactivity, and poor diet are associated with morbidity and premature death. Health promotion and primary prevention counseling, advice, and support by a primary care provider lead to behavior change attempts among patients. However, although physicians consider preventative health important, there is often a larger focus on symptom presentation, acute care, and medication review.

**Objective:**

This study evaluated the feasibility, adoption, and integration of the tablet-based Risk Factor Identification Tool (RFIT) that uses algorithmic information technology to support obtainment of patient risk factor information in primary care clinics.

**Methods:**

This is a pragmatic developmental evaluation. Each clinic developed a site-specific implementation plan adapted to their workflow. The RFIT was implemented in 2 primary care clinics located in Manitoba. Perceptions of 10 clinic staff and 8 primary care clinicians informed this evaluation.

**Results:**

Clinicians reported a smooth and fast transfer of RFIT responses to an electronic medical record encounter note. The RFIT was used by 207 patients, with a completion rate of 86%. Clinic staff reported that approximately 3%-5% of patients declined the use of the RFIT or required assistance to use the tablet. Among the 207 patients that used the RFIT, 22 (12.1%) smoked, 39 (21.2%) felt their diet could be improved, 20 (12.0%) reported high alcohol consumption, 103 (56.9%) reported less than 150 minutes of physical activity a week, and 6 (8.2%) patients lived in poverty. Clinicians suggested that although a wide variety of patients were able to use the tablet-based RFIT, implemented surveys should be tailored to patient subgroups.

**Conclusions:**

Clinicians and clinic staff positively reviewed the use of information technology in primary care. Algorithmic information technology can collect, organize, and synthesize individual health information to inform and tailor primary care counseling to the patients’ context and readiness to change. The RFIT is a user-friendly tool that provides an effective method for obtaining risk factor information from patients. It is particularly useful for subsets of patients lacking continuity in the care they receive. When implemented within a context that can support practical interventions to address identified risk factors, the RFIT can inform brief interventions within primary care.

## Introduction

Tobacco use, physical inactivity, and poor diet increase an individual’s risk of morbidity and premature death [1-7]. The World Health Organization estimates that 80% of cardiovascular diseases and 30% of cancer can be avoided with the implementation of health promotion and primary prevention strategies targeting smoking, diet, physical activity, and alcohol use [5,8]. The impact of these risk factors is increased by their relationship with mental illness [9] and the social determinants of health such as poverty [10].

Primary care is usually an individual’s initial point of contact with the health care system. Team-based primary care has gained support with the acceptance of models such as the Patient Centered Medical Home in the United States and Patient Medical Home in Canada [11,12]. These models include nurses and other providers who complement the care of a physician by providing a variety of services such as an initial assessment and follow-up with the patient (eg, test results, education resources) [10]. The role of the nurse and other providers may vary depending on the needs identified by the clinic as well as characteristics of the patient or appointment type. While providers consider preventative health important, they often fail to address primary prevention due to a focus on current symptoms, acute care, and medication concerns [10,13-16]. Primary care provides an important opportunity to identify risk behaviors and introduce primary prevention strategies [13,14,17-19]. Counseling, advice, and support from primary care clinicians increase awareness of potential behavior changes and are associated with patient’s attempting to change their behavior [13-24].

Algorithmic information technology can collect, organize, and synthesize individual health information to inform and tailor primary care counseling, positively impacting health outcomes and patient health behavior change [16,20-22,25-33]. It is also an efficient and useful means for assessing sensitive and stigmatizing information [24,32].

We developed an interactive computer-based application called the Risk Factor Identification Tool (RFIT) to support primary care clinics in obtaining risk factor information. In a previous study, we demonstrated that RFIT is a practical prevention tool in family practice [20]; however, it relied on computer-generated, printed patient responses that presented challenges during implementation [20]. Feedback received suggested the need for improved integration into the electronic medical record (EMR) to expedite availability of RFIT responses, provide a permanent and comprehensive record of risk behaviors, and enable personalized approaches to behavior change [20]. In this study, we aimed to evaluate the use of tablet-based technology with the immediate transfer of RFIT responses to the EMR. This pragmatic developmental evaluation describes the integration of the tablet-based RFIT into one rural Manitoba primary care clinic and one urban Manitoba primary care clinic.

## Methods

### The RFIT

The RFIT is a patient-centered assessment tool that applies motivational interviewing and health coaching modalities [22]. The RFIT collects basic demographic data from the patient and assesses patient risk behaviors (ie, physical activity, diet, smoking, and alcohol consumption) using previously validated tools [20,34-37]. In addition, the RFIT includes questions about self-perceived health and poverty (Multimedia Appendix 1). The RFIT uses a response-based algorithm. For example, the tool includes the CAGE [37] questions to assess alcohol dependency among patients who report alcohol consumption over the suggested age- and sex-based limits. The transtheoretical model used in the RFIT is widely accepted as a foundation for health behavior change and a basis for effective counseling approaches [23,30,38].

### Recruitment

A convenience sample of one rural clinic and one urban clinic was recruited in Manitoba, Canada. One clinic had a previous relationship with the research team. The medical director of the other clinic approached research staff at a conference about participating in research. The study team presented to interested primary care clinicians at each site, who then signed the information and consent forms. Participating clinicians offered the RFIT to patients attending a routine care appointment focused on health maintenance. Patients accepted the information and the consent form on the tablet prior to completing the RFIT and could stop the RFIT at any time.

### RFIT Implementation

Each site created a clinic-specific implementation plan that could be incorporated within their workflow. Nurses at the included clinics would often conduct a brief same-day assessment prior to the patient seeing the physician. Depending on the patient, this initial assessment may include documentation of height and weight, preappointment tests, and brief review of the patient’s concerns. Depending on clinic workflow, the patient may have completed RFIT before or after meeting with the nurse. After patients completed the RFIT, the responses were electronically transmitted as an encounter note into the EMR accessed by the physician using the Ocean App developed by CognisantMD [39] (Multimedia Appendix 2).

The participatory nature of this study allowed refinement and adjustment of the RFIT and EMR encounter note to meet the unique needs of each clinic. Ongoing feedback between clinic staff and the research team provided information regarding implementation progress and outcomes. The Ocean online platform provides user log and tablet audit reports to assess use and survey completion.

There were 3 focus groups held with a total of 10 clinic staff and 8 clinicians to discuss the RFIT. The clinic manager assisted the research team in arranging the lunch time focus groups. To accommodate clinic schedules, the first clinic held 2 focus groups, 1 for clinic staff and 1 for clinicians. The second clinic offered 1 focus group attended by both clinic staff and clinicians. Focus groups were led by 2 members of the research team. The majority of focus group participants were female and ranged in age from early 20s to mid-60s. Focus groups were attended by the clinic managers, physicians, nurses, and reception staff. The focus group addressed RFIT implementation, feasibility, and integration with questions focused on (1) experience with the tablet-based RFIT, (2) perceived value, (3) well-received features, (4) challenges experienced, (5) proposed solutions, (6) perception of increasing risk factor awareness, and (7) recommendations for expanded implementation of RFIT to other clinics. Consent was obtained prior to the focus groups. Focus groups were recorded and transcribed by the research team.

### Analysis

Qualitative results from clinicians and clinic staff presented details of RFIT feasibility, integration, and acceptance. Two team members who attended the focus groups reviewed the resulting transcripts. A preliminary coding dictionary was developed based on the focus group guide. Two researchers analyzed the transcripts from focus groups using the coding dictionary. Through consensus, team members generated common themes that emerged within each of the overarching categories (eg, implementation, feasibility, and integration). The implementation category had 1 theme focused on introduction of the tablet and RFIT to clinic processes. There were 4 themes within the feasibility category focused on well-received aspects of new technology, technology-related disruptions, establishing a new routine, and disruption in clinic workflow. Within the integration category, we describe 3 themes: information gain and clinical value of RFIT, patients’ reactions, and solutions to meet identified challenges (ie, tailoring the survey and adjustment to the process). Themes were shared with the clinic staff and clinicians to verify that themes matched participants’ experience(s). Focus group findings were supplemented with notes from virtual and in-person visits provided by the study team to the clinic to support implementation at each clinic.

Quantitative data including user logs, audit reports, and patient RFIT responses were recorded in Excel spreadsheets. RFIT responses included patient demographics, risk behaviors, self-perceived health, and self-reported low income (ie, do you find yourself running out of money to pay for food or shelter; do you have trouble paying for medications; do you receive any monthly benefits; do you have a clean and safe place to live). Descriptive statistics including frequency, mean, SD, and range describe the data. The Health Research Ethics Board at the University of Manitoba approved this study.

## Results

### Implementation

**Figure 1:**
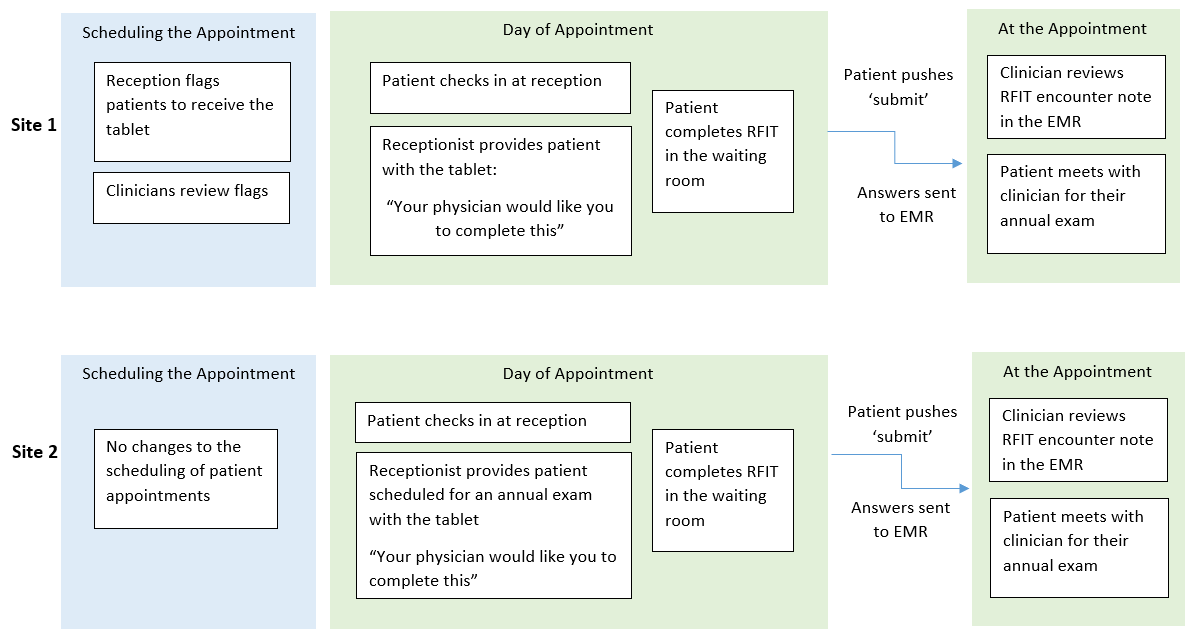
Each clinic designed their own Risk Factor Identification Tool (RFIT) implementation process to fit within the clinic workflow. EMR: electronic medical record.

Eight clinicians in 2 Manitoba clinics offered the tablet-based RFIT to patients attending routine care appointments at the primary care clinic for 6 months. There were 6 female and 2 male clinicians that used RFIT. Participating clinicians ranged in age from 28 years to 64 years. Although the study team provided implementation suggestions, each site developed their own implementation plan (Figure 1).

At the first site, reception staff added flags in the EMR for patients that were scheduled for routine care with 1 of the 3 participating clinicians. The clinician would review and, as appropriate, remove flags if the patient should not receive the RFIT. Staff at the clinic explained, “When the patient checks in at the clinic, a flag [in the EMR] will remind the front desk to provide them with a tablet.” At the second site, 5 clinicians participated using 2 tablets. The reception team provided the tablet, as available, to anyone arriving at the office for whom a routine visit was scheduled.

In total, there were 207 patients that started the RFIT, with a completion rate of 86% (179/207). Table 1 provides key characteristics from responses of patients who used the RFIT at each clinic. Overall, participant characteristics were similar in both sites. A notable exception was the number of respondents who were flagged by a CAGE question. Interestingly, the majority of patients from both sites chose not to respond to the self-reported low-income questions.

**Table 1:** Characteristics of patients that responded to the Risk Factor Identification Tool (RFIT) on the Ocean tablet (n=207).

Variable^a^		Site 1: rural clinic (n=116)	Site 2: urban clinic (n=91)	Missing responses
**Patient characteristics**				
	Age (years), mean (SD)	53 (16.8)	54 (15.1)	1
	Female patient, n (%)	83 (80.6)	62 (72.9)	20
	BMI, mean (SD)	26.6 (6.5)	28.4 (8.5)	31
**Health status**				
	Excellent, very good, or good self-perceived health, n (%)	91 (91.0)	69 (83.1)	25
	Self-perceived health unchanged from last year, n (%)	70 (71.4)	57 (69.5)	25
**Risk factors**				
	Self-reported smoker, n (%)	11 (10.9)	11 (13.6)	25
	Self-reported healthy diet, n (%)	80 (79.2)	65 (78.3)	23
	CAGE flag for alcohol consumption, n (%)	7 (7.5)	13 (17.3)	40
	Less than 150 minutes of physical activity, n (%)	56 (54.9)	47 (58.8)	26
	Employed, n (%)	56 (55.4)	50 (64.1)	28
	Low income, n (%)	5 (10.9)	1(3.6)	134

^a^Questions were not mandatory; patients could choose whether to answer a question.

### Assessing Feasibility

Feedback from the clinic staff and clinicians suggested there were benefits and challenges to both implementation strategies (Figure 1). Overall, the use of a tablet to facilitate the RFIT was well-received. However, reception staff mentioned that there were some technology-related disruptions and connectivity problems. When this occurred, the patient was unable to complete the RFIT. In addition, both clinics found there were some disruptions in clinic workflow. However, reception staff indicated that once they had established a routine, the use of the tablet increased (Table 2).

**Table 2:** Assessing feasibility themes with illustrative quotes from clinicians and clinic staff.

Themes and respondent roles		Illustrative quotes
**Well-received aspects of new technology**		
	Clinician	“*The tablet to EMR*^a^* connection is quick, with information appearing in the EMR immediately following the patient pressing the ‘submit’ or ‘quit’ buttons.*”
**Technology-related disruptions**		
	Reception	“*You type in the PHIN*^b^*, and then it says unable to connect...*”
**Disruption in clinic workflow**		
	Clinician	“*This avenue *[Ocean app]* for the RFIT*^c^* survey is preferential to paper. It increases its utility. However, you would need more tablets, and it still does slow down the clinic.*”
	Reception	“*Entering the tablet password and patient PHIN can be difficult if there is a long line at reception. If there was more… tablets and more physicians participating, it would be very difficult to set-up the tablet for each patient...*”
**Establishing a new routine**		
	Reception	“*Once we became more used to handing out the tablet and it became a habit, it really was not extra work administratively. It was easy in that sense.*”

^a^EMR: electronic medical record.

^b^PHIN: personal health information number.

^c^RFIT: Risk Factor Identification Tool.

The RFIT did require some adjustments to clinic workflow. On average, patients took 11 minutes to complete the RFIT. Clinic staff suggested older patients were initially flustered or anxious about using a tablet, and some patients found the RFIT lengthy. Both sites did mention that there were patients that did not complete the RFIT prior to their appointment.

Some patients were asked by their physician [if they could] complete the [RFIT] survey after their appointment… The physician can review the information before their next appointment.

Clinic staff

Physicians were reluctant to request patients arrive early for their appointment. There were very few patients that did not want to complete the tool. Reception explained:

Approximately 3%-4% of patients decline to complete the survey. Either they do not want their information to be entered on a tablet [due to privacy concerns], they do not know how to use a tablet, or there is a language barrier… Some patients felt questions in the survey… would be discussed with their physician...

Clinic staff

Older patients who come in for an annual physical will also be required to have some tests… Sometimes patients do not want to complete the survey if they have already completed other tests, they just want to see the doctor.

Clinic staff

### Assessing Integration

During the 6-month study period, patients completed the RFIT on 35.8% of the clinic days, with an average of 2 patients completing the RFIT each day it was administered. Clinic staff explained that routine care appointments are often scheduled for select days or times. Acute care appointments make up the majority of care provided, with only 1 or 2 routine care appointments scheduled per physician a couple days a week. Some clinic staff suggested tablet use might increase by providing, “*a dropdown list on the tablet...* [to] *select the physician and patient* [by name]...”

Clinicians felt that the tablet provided new information by creating a space to discuss alcohol and smoking. The summarized responses that appear in the EMR suggest topics to discuss with patients that may not be “*immediately visible or easily known, unless the patient is asked about it specifically… in that respect, RFIT was useful...*” Clinicians did not always review the encounter note created from RFIT responses, and neither of the sites continued to use the RFIT after the study. Additionally, a physician mentioned (Table 3), “*Filling out the survey on the tablet slows down the flow of the clinic, and information acquired as a result does not add value to the patient’s appointment or care provision.*”

**Table 3:** Assessing integration themes with illustrative quotes from clinicians and clinic staff.

Themes and respondent roles		Illustrative quotes
**Information gain and clinical value of RFIT^a^**		
	Clinician	“*The tablet enables discussions around areas such as alcohol and smoking. It allows GPs*^b^ *to gage a patient’s exercise levels and therefore starts a discussion around physical activity.*”
	Clinician	“*If you read the note while the patient is in the room, you can refer to their responses *[from RFIT]* to initiate the discussion.*”
	Clinician	“*I am intrigued by the Ocean app, but if the only survey I was using was RFIT, I would not spend the money...*”
	Clinician	“*I guess it assisted in the conversation on these topics... But our EMR*^c^* also prompts us to discuss many of these topics already. Patients also seem more engaged these days on their health and primary prevention. Patients did not use questions from RFIT as prompts or starting points for discussion *[with their clinician]...* The piece that is missing is, what do you do with this information? How do you help or what resources are available... You end up doing what you always would’ve done.*”
**Solutions to meet identified challenges (ie, tailoring the survey and adjustment to the process)**		
	Clinician	“*I could see this working well for other types of appointments and surveys. It would be very helpful for necessary* [mental health] *paper forms that have to be typed into the computer *[EMR]... [or for] *walk-in appointments...* [or] *to assess risk factors for prenatal [visits].*”
	Clinician	“*Nutrition flag might be more meaningful if you could say how poor the patient’s nutrition is compared to other patients. A comparative measure of nutrition... Or focusing on some specific areas such as ‘how often do you eat out’ and ‘where do you usually eat out’.*”
**Patients’ reactions**		
	Reception	“*There has been a good response to the tablet by patients. Patients have found no issues with the interface.*”
	Reception	“*Most patients were happy with something to occupy their time in the waiting room.*”

^a^RFIT: Risk Factor Identification Tool.

^b^GP: general practitioner.

^c^EMR: electronic medical record.

One site did continue to use Ocean for other surveys that are available and suggested that RFIT could be improved by tailoring the survey to the appointment type or using comparative measures that may be more meaningful to the patient (Table 3): “*you are in the bottom 10% for adequate nutrition*” [Clinician]. Overall, the clinic staff reported that most patients did not have concerns about using the tablet-based RFIT and most patients were happy to complete the survey in the waiting room (Table 3).

## Discussion

This study adds to the literature informing the utility of information technology to support primary care practice with a focus on preventative care. Our findings are useful to others exploring this potentially transformative approach to primary care service delivery. We address 3 critical components of this change in practice. First, we address the acceptance and usability of technology. Clinicians and clinic staff reported that the tablet and Ocean connection to the EMR provided a user-friendly interface. Clinicians, clinic staff, and patients that used RFIT ranged in age from young adults to seniors. In particular, RFIT was used by early-career, mid-career, and late-career clinicians that all reported benefits to the use of technology for the collection of risk factors. Similarly, other research has found computer-assisted assessments provide a useful, feasible tool for identifying health risks and are favorably reviewed by clinicians and patients of varying ages and demographics [21,32,40]. Approximately 3%-4% of patients required assistance or declined the use of the tablet. Psychosocial considerations and factors that may be subject to social desirability biases were particularly well-received due to their ability to inform individual patient counseling [32,33]. Further refining the BMI and nutrition questions on the RFIT can avoid duplication with what is currently available on the EMR and can make resulting notes more meaningful to initiate patient discussions.

Using this particular technology, RFIT responses are immediately transferred into an encounter note and are permanently recorded in the EMR. Reminders during a clinical visit can effectively prompt the primary care clinician to attend to a broad range of topics.[33] Similar to the Case Finding Health Assessment Tool (CHAT), we found that the RFIT was an efficient means for identifying risk factors [40]. However, some clinicians participating in this study did not review the RFIT responses regularly. Most participants preferred the direct tablet-to-EMR response transfer over a paper format. They reported less disruption to clinic workflow with minimal negative feedback from patients. To further decrease disruption to clinic workflow, further research should assess the use of email correspondence that can provide RFIT to patients prior to their appointment.

Second, we present a flexible, pragmatic approach to introduce a new workflow into primary care clinics. By adapting our tool and process to fit local needs and assessing both process and outcome indicators, we ensured rapid learning and enhanced utility of the resulting tool [25]. For example, during the study, adjustments were made to the location of the tablets in the clinic to facilitate increased use, and encounter notes were adapted to highlight key RFIT responses as requested by the clinicians. Traditional approaches would not enable adjustments during the research process [25]. As suggested by Ahmad et al [32], implementation strategies need to consider diversity in clinical settings and populations. Adjustment and preparation of the tool for implementation created a tool with a range of implementation options for a variety of primary care settings and patient populations. Implementation may include EMR prompts or summarized EMR input focused on topics for discussion.

Third, appropriately tailored tools can support acquisition of information to inform care. Primary care clinicians in the United States reported spending on average 16% of office visits on health counseling [13]. Katz et al [15] reported that 87%-89% of Canadian family physicians indicated they were comfortable counseling patients on risk behaviors, with smoking most commonly discussed (79%). In our study, 12% of patients indicated that they smoked. Physical inactivity was common; 56% of patients were not sufficiently active. Clinicians in our study reported less need for primary prevention tools for long-term patients. Long-term patients that attend routine care appointments are more likely to have risk factor documentation in the EMR and therefore may not require the RFIT. The RFIT may be better suited for new patients, patients with infrequent appointments, or patients experiencing life transitions, such as pregnancy. The literature supports an increasing need for low-cost, practical tools to address patient risk behaviors. Complementing risk factor identification with linkages to allied health professionals who are able to provide practical interventions can support primary care clinicians when providing primary prevention counseling and encourage the formation of healthy habits in patients [14,41-43].

### Limitations

This study represents 2 clinics and 8 primary care clinicians in Manitoba. Our findings are not based on a representative sample of primary care clinicians. This study only represents consenting patients at each of the clinics. Clinicians and patients that chose to participate in this study may have a greater interest in primary prevention or the use of technology. Participating clinicians may also be more likely to discuss risk factors with their patients. We purposely chose clinics located in both a rural and urban area of the province and with different patient populations to elicit a range of views. Similar responses across the 3 focus groups offered convergence in forming themes.

We look forward to continuing our research on primary prevention and how to best meet the needs of clinicians and patients. Future research might consider if the RFIT can inform the care of other providers at the clinic such as nurses as well as if tailoring the RFIT to specific patient demographics and situations such as prenatal care may better inform primary prevention counseling.

### Conclusions

Participants positively reviewed the use of the RFIT. Algorithmic information technology can collect, organize, and synthesize individual health information to inform and tailor primary care counseling to the patients’ context and readiness to change their behavior. With this information, the clinician can individualize health promotion and prevention activities to the patients’ social microcontext [17]. Ensuring accessibility of the tool, tailoring the tool to the appointment and patient, and making EMR notes meaningful for patient discussion can all enhance RFIT utility. The RFIT is a user-friendly tool that provides an effective method for obtaining risk factor information from patients. If implemented within a context that can support practical interventions to address identified risk factors, the RFIT can support brief interventions within primary care [15].

## Appendix

Multimedia Appendix 1 The Risk Factor Identification Tool (RFIT) questions.

Multimedia Appendix 2 The electronic medical record (EMR) encounter note created for the physician from the Risk Factor Identification Tool (RFIT) Responses.

## Abbreviations

CHAT: Case Finding Health Assessment Tool
EMR: electronic medical record
RFIT: Risk Factor Identification Tool
