# Supplementary material for: Using Information Technology to Assess Patient Risk Factors in Primary Care Clinics: Pragmatic Evaluation
Source: JMIR Form Res. 2021 Feb 2;5(2):e24382. doi: 10.2196/24382 (PMC7886616; doi:10.2196/24382)
Supplement: Multimedia Appendix 1 [file formative_v5i2e24382_app1.docx]

Appendix A: RFIT Computerized Risk Behaviour Assessment

9-Digit Personal Health identification Number (To be entered by front desk staff to link to patients EMR)

Screen 1 **–**

This computer program will ask you questions about your health behaviours. Your responses will be sent to your health care provider's computer. The care you receive from your health care provider will not be affected if you choose not to use this program or decide to quit the program.

**This questionnaire does not replace the need to inform your health care provider of any changes in your health status during the visit. Please inform him/her of any changes since your last visit.**

Screen 2-Consent

Your answers will be useful to support the care you receive. In addition, we are interested in using these answers for research to find out how different patient behaviours affect health and wellness. Once you have answered questions on this tablet, the information is coded for privacy and may be sent to a protected research database with your permission. It may also be linked to other research databases for future studies. Your personal information such as your name or health number will not be part of the research database. You can also choose to not have your information included in future research.

Will you allow your responses to be sent to the research database?

Yes / No

Screen 3: Instructions

Each question can be answered by touching the screen. Once you have answered the question(s) press the "Next" button. You can press "Previous" to return to the previous screen.

To begin the program, please press the "Next" button now...

1a-

Date of Birth

DAY MONTH YEAR (Compute Age)

Age _________ Go to question 3

1b –

Are you male or female?

Male

Female

Go to question 2a

2a –

What is your height?

_______cm

OR

_________ft _____inches

Go to question 2b

2b –

What is your present weight?

________kg

OR

________lbs.

RFIT calculates and reports BMI number

Your BMI is: ______

YES Go to question 3a

3a –

In general, would you say your health is:

Excellent

Very good

Good

Fair

Poor

Don’t know/Unsure

Go to 3b

3b –

Compared to one year ago, how would you rate your health in general now?
 Much better
 Somewhat better
 About the same
 Somewhat worse
 Much worse

Go to 4

4a- In the past 12 months, how many times have you consulted or visited a physician outside of this clinic?

More than 5 times

2-5 times

Once

No other visits

4b - Do you regularly take any medications (prescription or over-the-counter) that have not been prescribed by a physician at this clinic?

No / Yes

Go to 5

5 Do you see a dental professional regularly (i.e. yearly or more often)

No / Yes

In the past year have you lost weight? (yes/no)

My weight loss during the past year was: (Intentional (I meant to lose weight), unintentional)

How much weight have you lost in the past year? (less than 10lbs, 10-20 lbs, more than 20 lbs)

Go to 6a

6a-

Check the answer that best describes you at the present time:

I have never smoked - go to 9

I used to smoke but quit more than 10 years ago –go to 9

I used to smoke but quit more than 1 year ago - go to 9

I have quit smoking within the last year- go to 7

I have cut back on the amount I smoke - go to 7

I smoke regularly (i.e. daily, weekly, monthly or as a social smoker)-go to 8

7a-

What strategy(s) did you use to quit smoking? (Select all that apply)

Abrupt stop (cold turkey)

Acupuncture

Individualized counseling from a health professional

Nicotine replacement (gum, patch,)

Zyban or other

Hypnosis

Self-help manual

Gradual reduction

Formal cessation program (with classes, group discussion, etc)

Other Go to 9a

7b-

*Assessment of readiness for change*

Check off all the statements that apply to you:

I've got a lot of support to remain a non-smoker. (MAINTENANCE) go to 11

It is still too early to tell if I will be successful. (ACTION) go to 11

I need a boost to help me resist going back to smoking (ACTION) go to 11

I don’t think I could ever smoke another cigarette (MAINTENANCE) go to 11

8a-

*Assessment for readiness for change*

*Please indicate which of the statements best reflects how you feel about smoking:*

*I am not interested in quitting smoking (PRECONTEMPLATIVE )- go to 9*

*I am thinking about quitting smoking (CONTEMPLATIVE) - go to 8b*

*I’d like to discuss how I could quit smoking (PREPARATION) - go to 8b*

*I’ve already made some attempts to quit smoking (ACTION-)-go to 9*

8b-

How difficult would it be for you to stop smoking?

Very easy

Easy

Difficult

Very difficult

Go to 9

9-

Do you currently have a healthy diet?

Yes, for sure

I think so

Probably not

Unsure–

go to 10

10a-

In a typical day, how many servings of fruit and vegetables would you eat?

Female: 1-3 Male: 1-4

4-6 5-6

7-8 7-10

More than 8 More than 10

11a-

In a typical day, how many servings of grain products would you eat?

Female: 1-3 Male: 1-3

4-5 4-6

6-7 7-8

More than 7 More than 8

Go to 12a

12a-

In a typical day, how many servings of milk or alternates would you have?

Male or Female: Less than 2 Go to 12b

2-3 Go to 13a

More than 3 Go to 13a

12b-

Are you lactose intolerant?

YES

NO

Go to 13a

13a-

In a typical day, how many servings of meat or alternates would you eat?

Male or Female: Less than 2 Go to 13b

2- 3 Go to 14

More than 3 Go to 14

13b-

Are you a vegetarian?

YES

NO

Go to 14

14-

How often do you choose whole grain bread, crackers and/or high fiber cereal?

All the time

Usually

Sometimes

Never

Go to question 15

15-

In the past 7 days, how often did you eat breakfast (aprox. within 1 hour of rising)?

Everyday

Usually

Sometimes

Rarely

Never

Go to question 16

16

Which of the following best describes how you feel about your present weight?

Underweight
 Acceptable weight
 Some excess weight
 Overweight
 Obese

Go to 17

17-

*Assessment of readiness for change*

Which of the following statements describes how you feel about your diet:

I am not concerned about my dietary intake (PRECONTEMPLATIVE) Go to 21a

I have been thinking about what I could do to improve my diet (CONTEMP) Go to 20

I've made some changes toward a healthier diet (ACTION) Go to 21a

18-

How confident are you that you could make some changes to your daily diet?

Very confident

Somewhat confident

Mildly confident

Not at all confident

Go to 19

Alcohol Use

19a - In a typical week, how many alcohol drinks do you consume? (one drink is equivalent to a 12-oz beer, a 5oz glass of wine, or a drink with 1oz of liquor).

Female: More than 7 go to 19c Male: More than 14 go to 19c

Less than 7 go to 19b Less than 14 go to 19b

None go to 20a None go to 20a

19b - In a typical week, how many alcoholic drinks do you consume on one occasion? (one drink is equal to a 12oz beer, a 5oz glass of wine, or a drink with 1oz of liguor).

Female: More than 3 drinks on one occasion go to 19c

Less than 3 drinks on one occasion go to 20a

Male: More than 4 drinks on one occasion go to 19c

Less than 4 drinks on one go to 20a

19c - Please select all the statements that describe you:

I have felt guilty or bad after drinking

I have needed a drink first thing in the morning to get going

It is time to cut down or stop drinking

I get annoyed when someone criticizes how much I drink

None of the above

Go to 19d

19d - Identify the statement which best describes your feelings on your drinking:

I have read about and looked into ways to reduce my drinking

I wish I knew more about or could get advice about excess drinking

My attempts to cut back or quit drinking have not been successful but I am trying

I am prepared to reduce the amount I drink

I do not want to discuss my drinking

Go to 20a

20a-

Choose one week in the last month that you would say is typical or usual for you. From the following list of physical activities, select the activities you do during your typical or usual week.

Basketball or Volleyball

Squash or Racquetball

Soccer, Football, Rugby or Lacrosse

Hockey

Skiing or Snowboarding

Running

Skating or Rollerblading

Martial Arts

Boxing or Wrestling

Swimming

Go to 21b

If activity selected: (Question below will appear for each activity separately)

21b-

What is the TOTAL number of minutes per week you spend doing this activity?

|  |
| --- |

Go to 22a
22a-

Here is a second list of activities. Select the activities you do during your typical or usual week.

Household chores

Baseball or cricket

Dance or Aerobics

Bowling

Weightlifting or Circuit Training

Tennis or Badminton

Gardening/Lawn Care or Shoveling Snow

Cycling

Walking (for exercise)

Golf

Curling

Other activities

If activity selected Go to 22b (Questions below will appear for each activity separately)

22b-

When you are doing this activity, how hard are you breathing?

Heavy (I can barely talk) Go to 22c

Moderate (I can maintain a conversation) Go to 22d

Light (I can speak easily) Go to 23 (A or B determined by sum of minutes)

22c-

What is the TOTAL number of minutes per week you spend doing this Heavy breathing activity?

|  |
| --- |

Minutes
 Go to 22d

22d-

What is the TOTAL number of minutes per week you spend doing this moderate breathing activity?

|  |
| --- |

Minutes

23a - Other than the activities on the lists, are there physical activities you participated in during that week?

No (go to 24) /Yes (go to 23b)

23b - What other physical activities did you participate in? _____________

When you were doing this activity, how hard were you breathing

Heavy (I can barely talk) Go to 23c

Moderate (I can maintain a conversation) Go to 23d

Light (I can speak easily) Go to 24 (A or B determined by sum of minutes)

23c - What is the TOTAL number of minutes per week you spend doing this heavy breathing activity? ________

23d - What is the TOTAL number of minutes per week you spend doing this Moderate breathing activity?

Go to 24 (A or B determined by sum of minutes)

Total Score: number of minutes of physical activity in a typical or usual week.

*Assessment of readiness for change – based on whether 150 PA minutes are met*
(>150 minutes)

24A: Identify the statement that best applies to you:

1. I have to really work at maintaining my present level of activity
2. Regular physical activity has become part of my normal routine

Go to 26

OR…(if <150 minutes)

24B: Identify the statement that best applies to you:

1. I am not ready to make any changes to my physical activity right now
2. I’ve been thinking about how I could become more active
3. I’ve made some attempts to increase my activity

Go to 24c

24c-

How difficult would it be for you to increase your physical activity level?
 Very easy
 Easy
 Manageable
 Very difficult

Go to 25

25-

Does your physical health or pain limit your work or activities?
 Yes

No

Go to 26

26-

Does your activity level change according to the seasons?

Yes, I am more active in the summer.

Yes, I am more active in the winter.

No, I am equally active in the summer and winter.

Go to 27a

Safety

27a When riding a bicycle outdoors, how often do you wear a bicycle helmet?

Do not ride a bicycle outdoors

Every time you ride a bicycle

Most of the time

Rarely

Never

Go to 27b

27b Do you wear sunscreen with an SPF of 30 or higher when you are in the sunshine?

Most of the time

Sometimes

Rarely

Never

Go to 27c

27c How often do you wear a hat to protect against sun exposure?

Most of the time

Sometimes

Rarely

Never

If patient age ≥65 go to 28a

If patient age <65 go to 29

28a - Since last being seen by this doctor have you had a fall with an injury that caused you to limit your regular activities for at least one day or to go see a doctor?

No / Yes

Go to 28b

28b - Since last being seen by this doctor, have you been involved in more than 1 car crash or collision?

Go to 28c

28c Which of the following statements are true for you?

I have taken someone else’s medication

I sometimes miss a dose of my medication

I sometimes take more or less of my medication depending on how I feel

None of these statements apply to me

Employment

29a - Are you presently employed?

No (go to 29b) / Yes (go to 29c)

29b - Do find yourself running out of money to pay for food or shelter?

No / Yes

29c – Do you have trouble paying for medications?

29d – Do you receive any monthly benefits?

29e – Do you have a clean and safe place to live?

Questionnaire COMPLETE (Go to 30)

29c - Think about your workplace and any physical chemical or other potential causes of injury. Are you able to identify any potential hazards?

No / Yes

29d – How often do you protect yourself against potential hazards at your workplace?

Always

Usually

Sometimes

Never

Questionnaire COMPLETE (If patient agreed to participate in the study (above) Go to 30a)

Your answers are being sent to your health care provider.

30 You have been invited to take part in a research study. The information collected during this study will be used to increase your health care provider's ability to discuss risk factors at appointments. Can we call you to ask you about risk factor discussion that occurred during your appointment?

30a what is your first name?

30b What is you telephone number?

30c what is the best time of the day to call you (morning (9:00am – 12:00 pm), afternoon (1:00pm to 4:00pm), evening(7:00pm to 9:00pm))

Please return the tablet to the front desk. If you have any questions about this study, feel free to call Leanne Kosowan at 204-272-3086. This study has been approved by the University of Manitoba Health Research Ethics Board, that can be contacted at 204-789-3389.
