# Supplementary figures and images for: Using Information Technology to Assess Patient Risk Factors in Primary Care Clinics: Pragmatic Evaluation
Source: JMIR Form Res. 2021 Feb 2;5(2):e24382. doi: 10.2196/24382 (PMC7886616; doi:10.2196/24382)

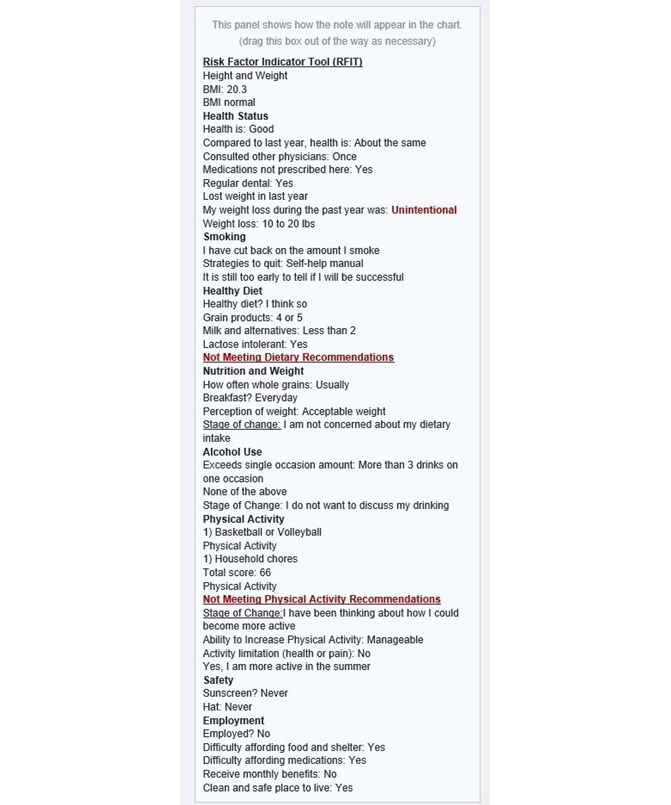

Supplement: Multimedia Appendix 2 [file formative_v5i2e24382_app2.png]
